# Predictive factors and the management of hyperglycemia in patients with acromegaly and Cushing’s disease receiving pasireotide treatment: *post hoc* analyses from the SOM230B2219 study

**DOI:** 10.3389/fendo.2024.1250822

**Published:** 2024-03-21

**Authors:** Ulla Feldt-Rasmussen, Marek Bolanowski, Shao-Ling Zhang, Yerong Yu, Przemysław Witek, Pramila Kalra, Noppadol Kietsiriroje, Andrea Piacentini, Alberto M. Pedroncelli, Susan L. Samson

**Affiliations:** ^1^ Department of Medical Endocrinology and Metabolism, Copenhagen University Hospital Rigshospitalet, Copenhagen, Denmark; ^2^ Institute of Clinical Medicine, Faculty of Health and Medical Sciences, Copenhagen University, Copenhagen, Denmark; ^3^ Department of Endocrinology, Diabetes and Isotope Therapy, Wroclaw Medical University, Wroclaw, Poland; ^4^ Sun Yat-Sen Memorial Hospital, Sun Yat-Sen University, Guangzhou, China; ^5^ West China Hospital, Sichuan University, Chengdu, China; ^6^ Department of Internal Medicine, Endocrinology and Diabetes, Medical University of Warsaw, Warsaw, Poland; ^7^ Department of Endocrinology, MS Ramaiah Medical College and Hospitals, Bengaluru, India; ^8^ Endocrinology and Metabolism Unit, Internal Medicine Department, Faculty of Medicine, Prince of Songkla University, Songkhla, Thailand; ^9^ Recordati SpA, Milan, Italy; ^10^ Recordati AG, Basel, Switzerland; ^11^ Departments of Medicine and Neurologic Surgery, Mayo Clinic, Jacksonville, FL, United States

**Keywords:** hyperglycemia, glucose intolerance, diabetes mellitus, pasireotide, acromegaly, Cushing’s disease, pituitary adenoma

## Abstract

**Introduction:**

Pasireotide, a somatostatin receptor ligand, is approved for treating acromegaly and Cushing’s disease (CD). Hyperglycemia during treatment can occur because of the drug’s mechanism of action, although treatment discontinuation is rarely required. The prospective, randomized, Phase IV SOM230B2219 (NCT02060383) trial was designed to assess optimal management of pasireotide-associated hyperglycemia. Here, we investigated predictive factors for requiring antihyperglycemic medication during pasireotide treatment.

**Methods:**

Participants with acromegaly or CD initiated long-acting pasireotide 40 mg/28 days intramuscularly (acromegaly) or pasireotide 600 μg subcutaneously twice daily during pre-randomization (≤16 weeks). Those who did not need antihyperglycemic medication, were managed with metformin, or received insulin from baseline entered an observational arm ending at 16 weeks. Those who required additional/alternative antihyperglycemic medication to metformin were randomized to incretin-based therapy or insulin for an additional 16 weeks. Logistic-regression analyses evaluated quantitative and qualitative factors for requiring antihyperglycemic medication during pre-randomization.

**Results:**

Of 190 participants with acromegaly and 59 with CD, 88 and 15, respectively, did not need antihyperglycemic medication; most were aged <40 years (acromegaly 62.5%, CD 86.7%), with baseline glycated hemoglobin (HbA_1c_) <6.5% (<48 mmol/mol; acromegaly 98.9%, CD 100%) and fasting plasma glucose (FPG) <100 mg/dL (<5.6 mmol/L; acromegaly 76.1%, CD 100%). By logistic regression, increasing baseline HbA_1c_ (odds ratio [OR] 3.6; *P*=0.0162) and FPG (OR 1.0; *P*=0.0472) and history of diabetes/pre-diabetes (OR 3.0; *P*=0.0221) predicted receipt of antihyperglycemic medication in acromegaly participants; increasing baseline HbA_1c_ (OR 12.6; *P*=0.0276) was also predictive in CD participants. Investigator-reported hyperglycemia-related adverse events were recorded in 47.9% and 54.2% of acromegaly and CD participants, respectively, mainly those with diabetes/pre-diabetes.

**Conclusion:**

Increasing age, HbA_1c_, and FPG and pre-diabetes/diabetes were associated with increased likelihood of requiring antihyperglycemic medication during pasireotide treatment. These risk factors may be used to identify those who need more vigilant monitoring to optimize outcomes during pasireotide treatment.

## Introduction

Acromegaly and Cushing’s disease are rare, often debilitating endocrine conditions that, if left untreated, are associated with considerable comorbidities and increased mortality (1, 2). Both conditions are most commonly caused by a benign pituitary adenoma that secretes growth hormone (GH) in cases of acromegaly and adrenocorticotropic hormone (ACTH) in cases of Cushing’s disease (3). Increased GH (and, consequently, insulin-like growth factor 1 [IGF-1]) or increased ACTH (leading to elevated cortisol) are associated with multiple underlying conditions, including impaired glucose tolerance and diabetes (4–7). Pasireotide, a second-generation somatostatin receptor ligand, is an effective long-term, pituitary-targeted medical treatment approved for patients with acromegaly (for whom surgery is not an option or has not been curative and who are inadequately controlled on treatment with another somatostatin analogue) (8–10) or Cushing’s disease (for whom pituitary surgery is not an option or has failed) (10–12). Pasireotide works by targeting four of the five somatostatin receptor (SSTR) subtypes, predominantly SSTR5 and SSTR2, all of which can be expressed by pituitary tumors (13). However, SSTR5 and SSTR2 also play important roles in blood glucose regulation; pancreatic beta cells, which secrete insulin, predominantly express SSTR5, and pancreatic alpha cells, which secrete glucagon, predominantly express SSTR2 (14). As shown in healthy volunteers, as well as in those with active acromegaly, pasireotide suppresses insulin secretion, with smaller reductions in glucagon secretion, resulting in elevated blood glucose; this is, at least in part, mediated by dampening of the incretin response (glucagon-like peptide 1 [GLP-1] and gastric inhibitory polypeptide [GIP]) (15, 16). Development or worsening of existing hyperglycemia is therefore an expected side effect of treatment with pasireotide, although it is not experienced by all patients (17). Addition of metformin, alone or in combination with other oral antihyperglycemic medications, was shown to control glucose elevations in most patients with acromegaly (17). Incretin-based therapies (eg dipeptidyl peptidase 4 [DPP-4] inhibitors [eg vildagliptin] and GLP-1 receptor agonists [eg liraglutide]) have also been shown to be an effective treatment option in managing pasireotide-associated hyperglycemia (18, 19). As such, with appropriate management, treatment-emergent hyperglycemic events rarely lead to treatment discontinuation (19), allowing the clinical benefit of pasireotide treatment to be achieved (8, 9, 11, 12) and sustained for many years (20).

The Phase IV B2219 trial was the first prospective study specifically designed to assess the efficacy of incretin-based therapy versus insulin in the management of pasireotide-associated hyperglycemia that is not fully controlled despite treatment with metformin or other non-incretin-based oral antidiabetic drugs (OADs) in participants with acromegaly or Cushing’s disease (21). A large proportion (41%) of participants in the B2219 study did not require any antihyperglycemic medication during treatment with pasireotide (21). An additional 18% were managed solely with metformin/other oral non-incretin-based medications, and 8% received insulin from baseline (with or without additional metformin during the study). Overall, 33% received antihyperglycemic medication in addition to metformin to manage hyperglycemia (21). This *post hoc* analysis examines patient characteristics that might predict those more or less likely to require initiation of or additional antihyperglycemic medications during pasireotide treatment. Furthermore, we also sought to investigate how insulin requirements may change throughout treatment with pasireotide. Together, we hope that these analyses will advance our knowledge of the proactive management of pasireotide-associated hyperglycemia.

## Materials and methods

### Participants

Outcomes from the B2219 trial (ClinicalTrials.gov: NCT02060383) have been published previously (21). Briefly, the trial enrolled adult participants with confirmed acromegaly or Cushing’s disease compliant with the country label to receive pasireotide. If participants were receiving pasireotide at screening, they had to be experiencing signs/symptoms of hyperglycemia to be enrolled. The study was conducted in accordance with the Declaration of Helsinki, with an independent ethics committee/institutional review board at each site approving the study protocol. All participants provided written informed consent before participation.

### Study design

The B2219 trial design has been previously described in detail (21); the four study phases are summarized in **Table 1**. Briefly, this was a multicenter, randomized, open-label, Phase IV study conducted at 43 sites comprising a core phase (≤16-week pre-randomization period followed by 16-week randomized treatment period) and an optional extension phase. Upon entering the pre-randomization period, all patients initiated intramuscular long-acting pasireotide 40 mg once/28 days (acromegaly) or subcutaneous pasireotide 600 μg twice daily (bid; Cushing’s disease). Titration of pasireotide dose was determined by the site investigator based on the biochemical response using local laboratory monitoring of IGF-1 or urinary free cortisol. Medication for hyperglycemia was initiated if self-monitored blood glucose (SMBG) levels were ≥126 mg/dL on three consecutive days. Participants who did not require antihyperglycemic medication, who were successfully managed with metformin alone, or who were receiving insulin from study baseline entered a non-randomized observational arm. Participants who developed hyperglycemia not manageable with metformin (SMBG ≥126 mg/dL on three consecutive days) were randomized to incretin-based therapy or insulin, administered in accordance with local prescribing information.

**Table 1:** Summary of the B2219 study design.

Period	Overview
1. Screening	• Washout period for participants receiving pasireotide at screening (or other medications, including antihyperglycemic medications)
2. Core pre- randomization (≤16 weeks)	• All participants initiated long-acting release pasireotide 40 mg intramuscularly once every 28 days (acromegaly) or pasireotide 600 μg subcutaneously twice daily (Cushing’s disease) • Pasireotide dose was adjusted based on biochemical response and tolerability • Metformin treatment could be initiated in participants with increased SMBG (≥126 mg/dL [≥7.0 mmol/L] on three consecutive days)
3. Core randomized treatment (~16 weeks)	• Participants with SMBG ≥126 mg/dL (≥7.0 mmol/L) on three consecutive days despite optimized treatment with metformin or other permitted OAD (or a contraindication to metformin) were randomized 1:1 to incretin-based therapy (sitagliptin [DPP-­4 inhibitor] followed by liraglutide [GLP-1 receptor agonist] and insulin rescue therapy, if needed) or insulin
4. Optional extension	• Non-randomized participants could continue receiving pasireotide and antihyperglycemic therapy at the investigator’s discretion

### Assessments and statistical analyses

Glycated hemoglobin (HbA_1c_) and fasting plasma glucose (FPG) were measured at a central laboratory. HbA_1c_ was recorded at screening, at baseline, once a month throughout the core study (including before starting rescue therapy), and every 8 weeks during the extension. FPG was monitored every 2 weeks during the core study. During the core phase, participants also self-monitored plasma glucose daily with a glucometer, reviewed by the investigator at each visit. During the extension, FPG was monitored every 4 weeks. All data analyses were performed separately for participants with acromegaly and those with Cushing’s disease. Participants were evaluated according to whether they received therapy for hyperglycemia during pasireotide treatment in the core pre-randomization period (mostly metformin, alone or in addition to insulin received from baseline). A logistic-regression analysis of predictive factors was conducted. A backward method for selecting the predictors was applied, with a threshold of *P ≤* 0.2 for remaining in the reduced (final) model. The criterion of –2 log likelihood was also applied to identify the reduced model that best fits the data. In the full model, age, body mass index (BMI), baseline HbA_1c_, baseline FPG, years with disease, baseline GH, and baseline IGF-1 (x upper limit of normal) were assessed as quantitative independent variables (evaluated by the model in terms of per unit increase). History of diabetes (pre-diabetes and diabetes vs normal glucose tolerance) and previous treatments received for acromegaly/Cushing’s disease (yes vs no) were assessed as qualitative independent variables. *P* values were derived from the type III analysis of effects and are shown for the overall effect of each predictor.

Hyperglycemia was defined as SMBG ≥126 mg/dL (≥7.0 mmol/L) on three consecutive days. All blood samples for assessment of FPG and HbA_1c_ at each visit were taken after an overnight fast and before administration of pasireotide. Diabetes was defined as participants taking antidiabetic medication, prior history of diabetes, or HbA_1c_ ≥6.5% (≥48 mmol/mol) and/or FPG ≥126 mg/dL (≥7.0 mmol/L) at two separate visits. Pre-diabetes was defined as participants with FPG ≥100 mg/dL (≥5.6 mmol/L) and/or HbA_1c_ 5.7–<6.5% (39–<48 mmol/mol). Normal glucose tolerance was defined as participants not qualifying as having diabetes or pre-diabetes, with FPG <100 mg/dL (<5.6 mmol/L) and HbA_1c_ <5.7% (<39 mmol/mol).

Hyperglycemia-related adverse events (AEs) were continually assessed and defined using Medical Dictionary for Regulatory Activities v21.0 and graded according to Common Terminology Criteria for Adverse Events (CTCAE) v4.03; relationship to study drug was assessed by the investigator.

## Results

### Participant demographics and disease history

**Figure 1:**
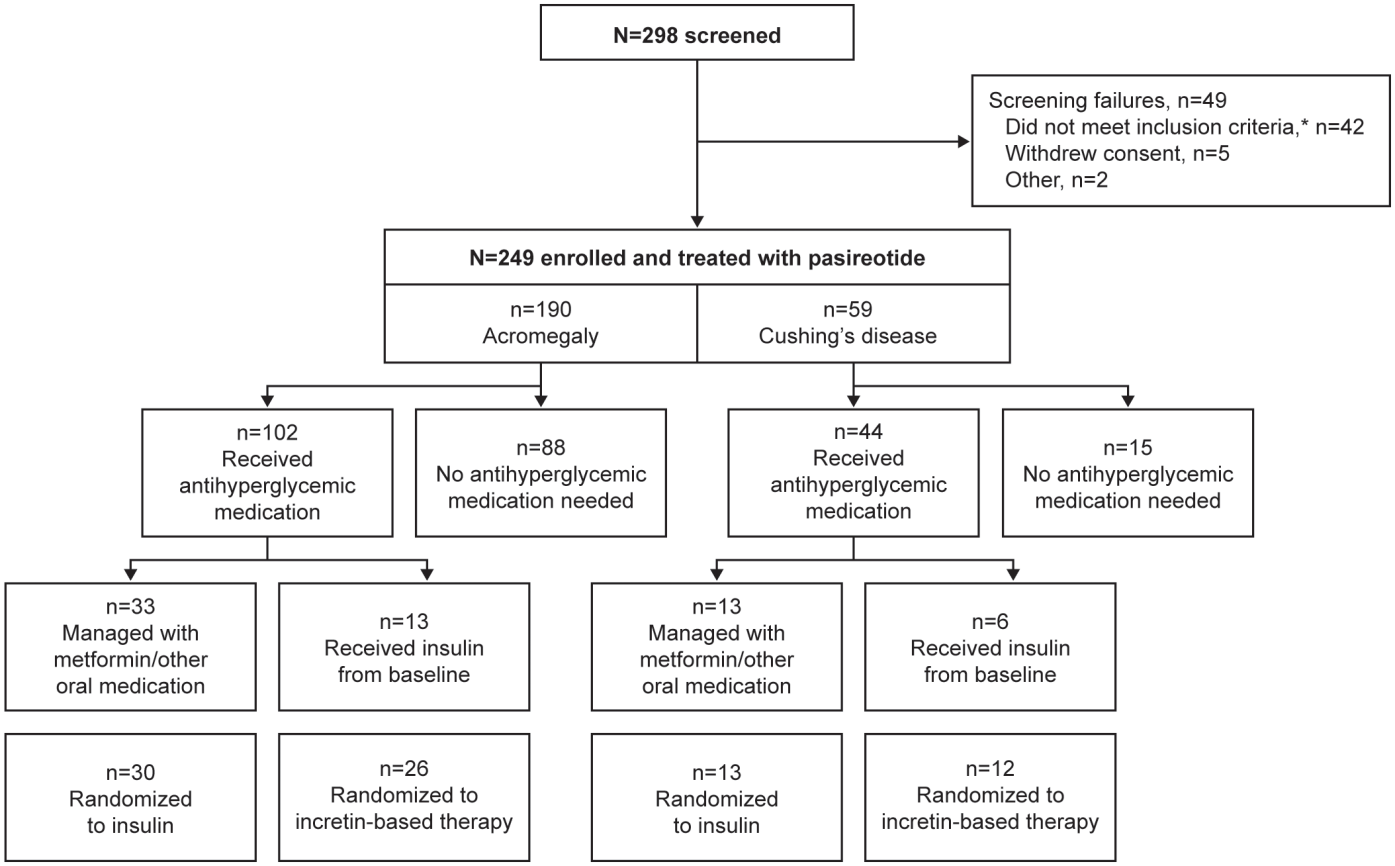
Participant disposition during the core phase of the study according to whether antihyperglycemic medication was received. *Includes unacceptable test procedure results, laboratory values, past medical history or concomitant medications.

In total, 190 participants were enrolled with acromegaly and 59 were enrolled with Cushing’s disease; 102/190 (53.7%) and 44/59 (74.6%) received antihyperglycemic medication during the pre-randomization period, respectively (**Figure 1**). Four participants with acromegaly received pasireotide prior to starting the study (for 2.4, 5.6, 10.2, and 70.8 months, respectively), as well as one participant with Cushing’s disease (for 24.2 months); all underwent the prerequisite washout of pasireotide prior to starting the pre-randomization period (≥3 months [long acting] or 1 week [twice-daily formulation]).

Participants (with acromegaly or Cushing’s disease) who received antihyperglycemic medication were generally older (mean age >40 years) and had higher baseline FPG and HbA_1c_ levels, and the majority were diagnosed with diabetes or pre-diabetes prior to starting the study (**Tables 2**, **3**).

**Table 2:** Baseline demographics and characteristics in participants with acromegaly.

	Received antihyperglycemic medication n=102*	No antihyperglycemic medication needed n=88*	All participants n=190*
**Mean age, years (SD)**	46.1 (12.9)	38.5 (10.8)	42.5 (12.5)
Min−max	21−79	22−66	21−79
**Female:male, n (%)**	54:48 (52.9:47.1)	35:53 (39.8:60.2)	89:101 (46.8:53.2)
**Race, n (%)**			
Asian	50 (49.0)	45 (51.1)	95 (50.0)
Caucasian	44 (43.1)	31 (35.2)	75 (39.5)
Other (including black and native American)	8 (7.8)	12 (13.6)	20 (10.5)
**Mean BMI, kg/m^2^ (SD)**	29.1 (5.1); n=101	28.3 (5.5); n=86	28.8 (5.3); n=187
Min−max	19.8−44.6	19.0−47.2	19.0−47.2
**Mean time to first pasireotide dose in the study since diagnosis, months (SD)**	66.8 (71.5)	56.2 (58.1)	61.9 (65.7)
Min−max	1.0−387.0	0.0−322.0	0.0−387.0
**Previous treatment for acromegaly, n (%)**	97 (95.1)	87 (98.8)	184 (96.8)
**Previous surgery, n (%)**	81 (79.4)	82 (93.2)	163 (85.8)
**Previous medication, n (%)**	74 (72.5)	69 (78.4)	143 (75.3)
**Previous pituitary irradiation, n (%)**	25 (24.5)	29 (33.0)	54 (28.4)
**Mean time since last irradiation, months (SD)**	57.4 (77.2)	38.3 (47.7)	47.2 (63.2)
Min–max	4.0−352.0	3.0−248.0	3.0−352.0
**Mean baseline HbA_1c_, % [mmol/mol] (SD)**	6.24 [45] (1.1); n=102	5.43 [36] (0.3); n=87	5.87 [41] (0.9); n=189
Min–max	4.6−10.6 [27–92]	4.5−6.0 [26–42]	4.5−10.6 [26–92]
**Baseline HbA_1c_ category, n (%)**			
<5.7% (<39 mmol/mol)	27 (26.5)	63 (71.6)	90 (47.4)
5.7–<6.5% (39–<48 mmol/mol)	49 (48.0)	24 (27.3)	73 (38.4)
6.5–<8% (48–<64 mmol/mol)	18 (17.6)	0	18 (9.5)
≥8% (≥64 mmol/mol)	8 (7.8)	0	8 (4.2)
**Mean baseline FPG, mg/dL [mmol/L] (SD)**	113.9 [6.3] (34.6); n=101	93.4 [5.2] (8.3); n=88	104.4 [5.8] (27.8); n=189
Min–max	60.0−295.4 [3.3–16.4]	70.3−120.7 [3.9–6.7]	60.0−295.4 [3.3–16.4]
**Baseline FPG category, n (%)**			
<100 mg/dL (<5.6 mmol/L)	36 (35.3)	67 (76.1)	103 (54.2)
100–<126 mg/dL (5.6–<7.0 mmol/L)	43 (42.2)	21 (23.9)	64 (33.7)
≥126 mg/dL (≥7.0 mmol/L)	22 (21.6)	0	22 (11.6)
**Baseline glycemic status, n (%)**			
Diabetes	57 (55.9)	0	57 (30.0)
Pre-diabetes	33 (32.4)	34 (38.6)	67 (35.3)
Normal glucose tolerance	12 (11.8)	54 (61.4)	66 (34.7)

*Unless otherwise specified. SD, standard deviation.

The gray shading is to separate blocks of data so that it is easier to read.

**Table 3:** Baseline demographics and characteristics in participants with Cushing’s disease.

	Received antihyperglycemic medication n=44*	No antihyperglycemic medication needed n=15*	All participants n=59*
**Cushing’s disease status, n (%)**			
*De novo*	5 (11.4)	4 (26.7)	9 (15.3)
Persistent/recurrent	39 (88.6)	11 (73.3)	50 (84.7)
**Mean age, years (SD)**	44.5 (14.5)	33.9 (12.8)	41.8 (14.7)
Min−max	18−72	21−70	18−72
**Female:male, n (%)**	36:8 (81.8:18.2)	12:3 (80.0:20.0)	48:11 (81.4:18.6)
**Race, n (%)**			
Asian	10 (22.7)	4 (26.7)	14 (23.7)
Caucasian	27 (61.4)	9 (60.0)	36 (61.0)
Other (including black and native American)	7 (15.9)	2 (13.3)	9 (15.3)
**Mean BMI, kg/m^2^ (SD)**	32.8 (8.9); n=43	32.0 (6.7); n=15	32.6 (8.3); n=58
Min−max	19.6−60.1	20.6−42.2	19.6−60.1
**Mean time to first pasireotide dose in the study since diagnosis, months (SD)**	67.8 (70.4)	36.2 (38.0)	59.8 (64.9)
Min−max	1.0−332.0	1.0−147.0	1.0−332.0
**Previous treatment for Cushing’s disease, n (%)**	37 (84.1)	10 (66.7)	47 (79.7)
**Previous surgery, n (%)**	40 (90.9)	11 (73.3)	51 (86.4)
**Previous medication, n (%)**	28 (63.6)	10 (66.7)	38 (64.4)
**Previous pituitary irradiation, n (%)**	13 (29.5)	4 (26.7)	17 (28.8)
**Mean time since last irradiation, months (SD)**	60.4 (50.6)	15.3 (11.4)	49.8 (48.3)
Min–max	8.0−161.0	4.0−29.0	4.0−161.0
**Mean baseline HbA_1c_, % [mmol/mol] (SD)**	6.4 [46] (0.8); n=43	5.5 [37] (0.4); n=15	6.2 [44] (0.8); n=58
Min–max	5.0−8.2 [31–66]	4.4−6.0 [25–42]	4.4−8.2 [25–66]
**Baseline HbA_1c_ category, n (%)**			
<5.7% (<39 mmol/mol)	8 (18.2)	10 (66.7)	18 (30.5)
5.7–<6.5% (39–<48 mmol/mol)	18 (40.9)	5 (33.3)	23 (39.0)
6.5–<8% (48–<64 mmol/mol)	16 (36.4)	0	16 (27.1)
≥8% (≥64 mmol/mol)	1 (2.3)	0	1 (1.7)
**Mean baseline FPG, mg/dL [mmol/L] (SD)**	111.2 [6.2] (32.5)	85.5 [4.7] (6.9)	104.7 [5.8] (30.4)
Min–max	79.3−262.0 [4.4–14.5]	70.3−97.3 [3.9–5.4]	70.3−262.0 [3.9–14.5]
**Baseline FPG category, n (%)**			
<100 mg/dL (<5.6 mmol/L)	21 (47.7)	15 (100.0)	36 (61.0)
100–<126 mg/dL (5.6–<7.0 mmol/L)	14 (31.8)	0	14 (23.7)
≥126 mg/dL (≥7.0 mmol/L)	9 (20.5)	0	9 (15.3)
**Baseline glycemic status, n (%)**			
Diabetes	30 (68.2)	0	30 (50.8)
Pre-diabetes	9 (20.5)	5 (33.3)	14 (23.7)
Normal glucose tolerance	5 (11.4)	10 (66.7)	15 (25.4)

*Unless otherwise specified.

The gray shading is to separate blocks of data so that it is easier to read.

### Pasireotide dosing

Average median pasireotide dose (min–max) during the pre-randomization period (up to week 16) was 40 mg/28 days (20–60 mg/28 days) and 1200 µg bid (600–1200 µg bid) in participants with acromegaly and Cushing’s disease, respectively.

Median (min–max) duration of exposure to long-acting pasireotide in participants with acromegaly was 3.7 (0.9–8.0) months, and that to subcutaneous pasireotide was 3.7 (0.0–6.8) months, in the core phase of the study (including the pre-randomization and randomized periods).

### Predictive factors for requiring antihyperglycemic medication during initiation of pasireotide treatment

**Figure 2:**
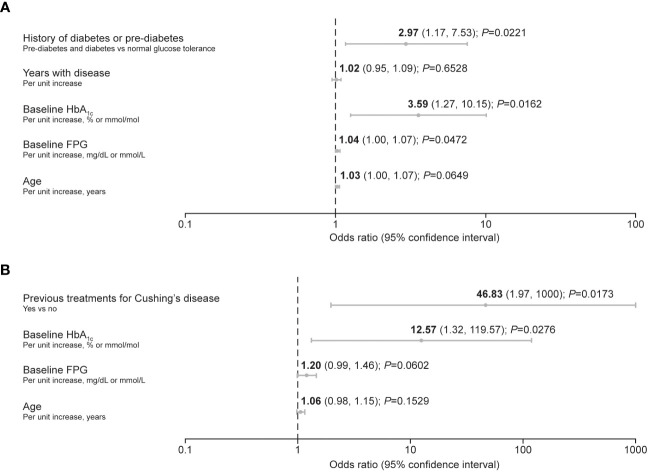
Forest plots summarizing predictive factors for the requirement of antihyperglycemic medication after initiation of pasireotide treatment in participants with **(A)** acromegaly and **(B)** Cushing’s disease. A backward method for selecting the predictors was applied, with a threshold of *P ≤* 0.2 for remaining in the reduced (final) model. The criterion of –2 log likelihood was also applied to identify the reduced model that best fits the data.

Predictive factors associated with the requirement for antihyperglycemic medication after initiation of pasireotide during the pre-randomization period of the study (≤16 weeks) were identified using logistic regression. In participants with acromegaly, increasing baseline HbA_1c_ and FPG, as well as history of diabetes/pre-diabetes, were identified as predictive factors (**Figure 2A**). In participants with Cushing’s disease, increasing baseline HbA_1c_ was also identified as a predictive factor, alongside receipt of previous treatment for Cushing’s disease (**Figure 2B**).

### Changes in glycemic variables by baseline diabetes status

**Figure 3:**
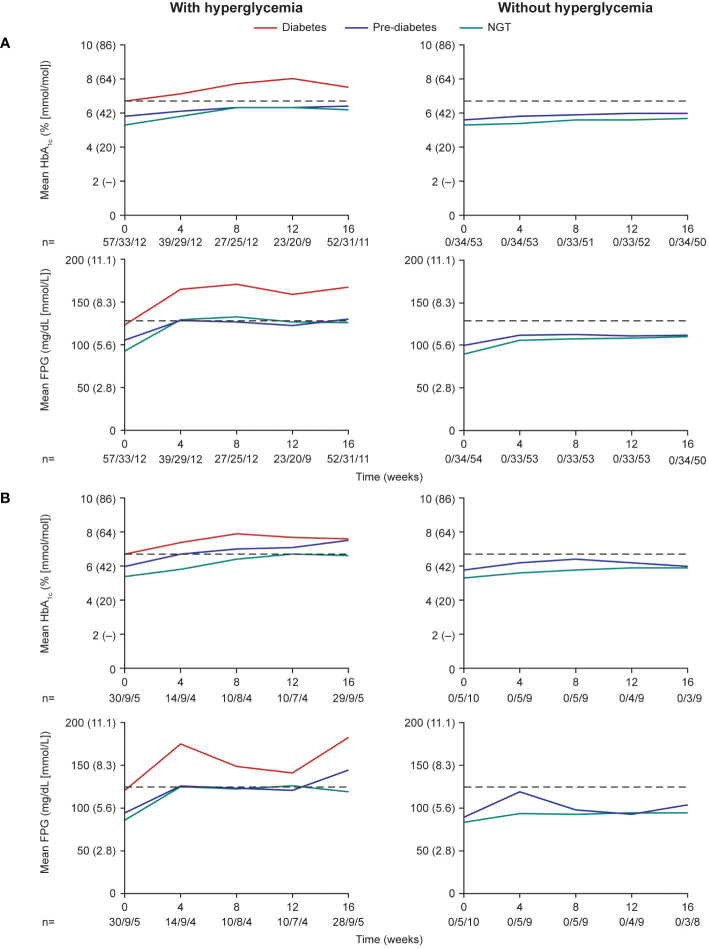
Mean HbA_1c_ and FPG levels over the first 16 weeks (pre-randomization period) in participants with **(A)** acromegaly and **(B)** Cushing’s disease. Numbers below the horizontal axes refer to numbers of participants with diabetes/pre-diabetes/NGT. Dashed lines are at 6.5% (48 mmol/mol) for HbA_1c_ and 126 mg/dL (7.0 mmol/L) for FPG. NGT, normal glucose tolerance.

During the pre-randomization period (≤16 weeks), mean HbA_1c_ and FPG levels increased after initiation of pasireotide in participants who received antihyperglycemic medication in both participants with acromegaly (**Figure 3A**) and participants with Cushing’s disease (**Figure 3B**). The trend for increasing HbA_1c_ and FPG levels was more notable in participants with diabetes or pre-diabetes at baseline than in those with normal glucose tolerance.

**Figure 4:**
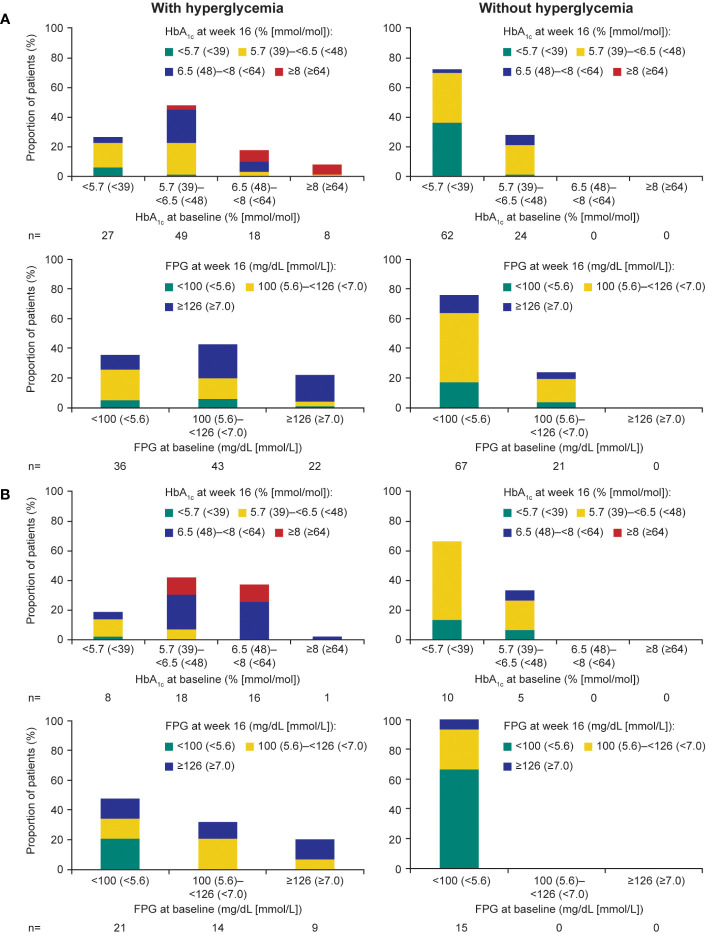
Shift from baseline to last post-baseline HbA_1c_ and FPG during the 16­week pre-randomization period in participants with **(A)** acromegaly and **(B)** Cushing’s disease.

During the ≤16-week pre-randomization period, participants who did not receive antihyperglycemic medication largely remained with low/normal HbA_1c_ and FPG levels in both those with acromegaly (**Figure 4A**) and those with Cushing’s disease (**Figure 4B**). Of participants who received antihyperglycemic medication, a larger proportion had higher HbA_1c_ and FPG levels at baseline than those who did not require antihyperglycemic medication (**Figure 4**). There was also evidence for a larger proportion of participants experiencing worsening HbA_1c_ and FPG (ie moved from a lower to a higher category during the ≤16-week pre-randomization period).

### Insulin use over time

In the participants randomized to receive insulin (n=43), mean (min–max) insulin dose increased from 9.0 (2.0–20.0) IU/day at randomization to 18.7 (4.0–50.0) IU/day at the end of the study; mean change from baseline was +9.7 (–4.0 to 38.0) IU/day. In participants with acromegaly (n=30), mean (min–max) insulin dose increased from 8.3 (2.0–20.0) to 16.7 (4.0–50.0) IU/day; mean change from baseline was +8.4 (–4.0 to 38.0) IU/day. In participants with Cushing’s disease (n=13), mean (min–max) insulin dose increased from 10.8 (8.0–15.0) to 23.4 (10.0–48.0) IU/day; mean change from baseline was +12.6 (0–38.0) IU/day. Median (min–max) duration of exposure to insulin in all participants randomized to insulin was 3.7 (1.4–4.3) months (21).

### Safety profile

During the pre-randomization period, grade 3/4 hyperglycemia-related AEs (including preferred terms: hyperglycemia, diabetes mellitus, impaired fasting glucose, increased blood glucose, increased HbA_1c_, type 2 diabetes mellitus, inadequate control of diabetes mellitus, and glycosuria) were infrequent and mostly occurred in participants with diabetes at baseline (**Tables 4**, **5**). In general, hyperglycemia AEs judged by the investigator to be related to treatment were reported irrespective of diabetes status at baseline.

**Table 4:** Incidence and severity of hyperglycemia-related AEs in participants with acromegaly receiving pasireotide, according to diabetes status.

	Diabetes (n=57)	Pre-diabetes (n=67)	Normal glucose tolerance (n=66)	All acromegaly participants (n=190)
**At least one hyperglycemia-related AE, n (%)**	22 (38.6)	37 (55.2)	32 (48.5)	91 (47.9)
Grade 3–4, n (%)	12 (21.1)	1 (1.5)	1 (1.5)	14 (7.4)
**Treatment-related hyperglycemia-related AE, n (%)**	19 (33.3)	33 (49.3)	32 (48.5)	84 (44.2)

**Table 5:** Incidence and severity of hyperglycemia-related AEs in participants with Cushing’s disease receiving pasireotide, according to diabetes status.

	Diabetes (n=30)	Pre-diabetes (n=14)	Normal glucose tolerance (n=15)	All Cushing’s disease participants (n=59)
**At least one hyperglycemia-related AE, n (%)**	15 (50.0)	11 (78.6)	6 (40.0)	32 (54.2)
Grade 3–4, n (%)	7 (23.3)	0	0	7 (11.9)
**Treatment-related hyperglycemia AE, n (%)**	9 (30.0)	10 (71.4)	6 (40.0)	25 (42.4)

During the first 16 weeks of the study (pre-randomization period), few participants required pasireotide dose reductions or interruptions because of hyperglycemia-related AEs: seven (3.7%) with acromegaly and six (10.2%) with Cushing’s disease. Of these 13 participants, 12 had diabetes or pre-diabetes at baseline. Two participants permanently discontinued because of hyperglycemia-related AEs (one documented as hyperglycemia and one as increased HbA_1c_), both with acromegaly and both with diabetes at baseline. Concomitant antihyperglycemic medication was required in 50 (26.3%) participants with acromegaly and 18 (30.5%) with Cushing’s disease; of these 68 participants, 52 had diabetes or pre-diabetes at baseline.

## Discussion

B2219 was the first trial to evaluate prospectively the management of pasireotide-associated hyperglycemia in a large number of patients with acromegaly or Cushing’s disease (21). Glucose metabolism disorders are common in patients with these endocrine disorders, with a high prevalence of complications such as impaired glucose tolerance and diabetes (4–7). As shown in other pivotal Phase III studies of pasireotide in patients with acromegaly or Cushing’s disease (9, 11), the majority of patients entering the B2219 study had diabetes or pre-diabetes (65.3% with acromegaly and 74.6% with Cushing’s disease). As hyperglycemia is a known side effect of treatment with pasireotide, patients can be appropriately monitored and managed (10). Previous data also highlight that if hyperglycemia does occur, it is most likely during the first 3 months of treatment with pasireotide (9, 22) and is reversible upon pasireotide discontinuation (17). However, it remains that some patients are more or less likely to require antihyperglycemic medications; therefore, understanding predictive patient characteristics is of interest.

In the present study, not all participants required antihyperglycemic medication. In total, 53.7% of participants with acromegaly and 74.6% of participants with Cushing’s disease received antihyperglycemic medication during the pre-randomization phase, mostly metformin, either alone or in addition to insulin received from baseline. Of these participants, approximately half (if eligible) were then randomized to receive additional/alternative antihyperglycemic medication in the form of incretin-based therapy or insulin. The participants who received antihyperglycemic medication in the 16 weeks following initiation of pasireotide were generally older (mean age >40 years), had higher baseline HbA_1c_ (>6.5% [>48 mmol/mol]) and FPG levels (>100 mg/dL [>5.6 mmol/L]), and were diagnosed with diabetes or pre-diabetes. The logistic-regression analysis also identified increasing baseline HbA_1c_ and FPG, as well as a history of diabetes/pre-diabetes, as risk factors for requiring antihyperglycemic medication in participants with acromegaly; notably, elevated baseline HbA_1c_ and a history of diabetes/pre-diabetes were associated with a threefold increase in risk. In participants with Cushing’s disease, increasing HbA_1c_ was also identified as a risk factor along with receipt of previous treatment, although the majority of participants with Cushing’s disease had received prior treatments (80%). Both mean HbA_1c_ and FPG levels increased steadily during the first 8–12 weeks of the study in participants who received antihyperglycemic medication, notably those with diabetes/pre-diabetes. *Post hoc* and exploratory analyses from other clinical trials of pasireotide in patients with acromegaly (C2305 and C2402 [PAOLA]) have also suggested that hyperglycemia during pasireotide treatment was less frequent in patients with lower age (<40 years, C2402; <30 years, C2305) and normal glucose tolerance (17, 23). As such, pre-treatment glucose status may be a particularly useful predictor of the development of pasireotide-associated hyperglycemia. History of hypertension or dyslipidemia at baseline has also been identified as a predictive factor (17); however, data were not available in the present study.

Interestingly, in the participants randomized to receive insulin in addition to or instead of metformin in the present study, dose requirements increased considerably in a relatively short period of time.

Hyperglycemia-related AEs were more frequently reported in participants with diabetes or pre-diabetes at baseline, and serious AEs related to hyperglycemia were infrequent. Our results highlight and support previous findings that hyperglycemia-related AEs rarely require treatment interruption or discontinuation (19), emphasizing that when hyperglycemia does occur, it is manageable. Several treatment guidelines and expert recommendations also exist on the management of hyperglycemia, which, although focused on patients with Cushing’s disease, can be extrapolated to those with acromegaly (24, 25). Together with the findings from the B2219 study and the results presented herein, patients can be appropriately managed on an individual basis to ensure continued treatment with pasireotide, which can provide prolonged maintenance of biochemical control and improve clinical symptoms (20). For most patients who develop hyperglycemia, initiation of medical therapy with metformin alone is sufficient, followed by staged treatment with incretin-based therapies and insulin, as required, to achieve and maintain glycemic control (21, 24). Furthermore, hyperglycemia may also be managed with dietary modification, exercise and education (24). As clinical experience with pasireotide is increasing, including long-term follow-up studies and real-world experience (26–28), management of hyperglycemia is also improving.

There is increasing evidence that a personalized medical treatment approach based on patient-specific clinical and tumor characteristics can improve outcomes in patients with acromegaly (29–31). As well as identifying which patients are more likely to respond to different medical therapies, our findings contribute to optimizing patient management during medical treatment, which will be useful in clinical practice to tailor therapeutic approaches.

We acknowledge the limitations of these *post hoc* analyses as they are descriptive in nature. Findings in participants with Cushing’s disease may be limited by the relatively small number of participants in this group compared with those with acromegaly. Levels of GH/IGF-1 and urinary free cortisol were measured but were used only to guide therapeutic decisions locally; they were not recorded as part of the study design, so no comment can be made on the impact of disease control on the need for antihyperglycemic medication. Furthermore, these analyses did not consider the occurrence/recurrence of hyperglycemia that may arise with long-term pasireotide treatment; to this end, further investigation is still warranted.

## Conclusion

Individual patient characteristics are useful indicators of whether patients are more or less likely to develop hyperglycemia during treatment with pasireotide. Notably, increased age, HbA_1c_ and FPG levels, as well as a previous diagnosis of diabetes or pre-diabetes, should be considered as potential predictive factors. These factors may be used to identify patients who require more vigilant, proactive monitoring and early intervention to ensure continued treatment with pasireotide and optimal outcomes.

## Data availability statement

The raw data supporting the conclusions of this article will be made available by the authors, without undue reservation.

## Ethics statement

The studies involving humans were approved by an independent ethics committee/institutional review board at each site. The studies were conducted in accordance with the local legislation and institutional requirements. The participants provided their written informed consent to participate in this study.

## Author contributions

All academic investigators enrolled patients in the study. Data were collected by investigators using the funder’s data management systems and analyzed by the funder’s statistical team. All authors contributed to the article and approved the submitted version.
